# Cohesion at First Sight. Experiences from Group Work in the BOJE Mental Health Support Programme (Colours) for LGBTQIA+ People in Croatia

**DOI:** 10.1192/j.eurpsy.2026.10915

**Published:** 2026-07-31

**Authors:** A. Vuk, J. Hubler, I. Barun, L. Mijalkovic, M. Supe, J. Pravdic, V. V. Dogas, V. Vercevic, V. Grosic

**Affiliations:** 1 Department of Integrative Psychiatry; 2 Addiction Day Hospital; 3 Eating Disorders Day Hospital; 4 Department of Psychotherapeutic Treatment; 5 BOJE, University Psychiatric Hospital Sveti Ivan, Zagreb, Croatia

## Abstract

**Introduction:**

Minority stress remains a central factor in the mental health of sexual and gender minorities often resulting in elevated distress, anxiety, depression, suicidality, and substance misuse (Moorhead, B., Lewis, H.K. & Arnull, L., 2024.). Building on insights from previous research, the Mental Health Support Programme BOJE (Colours) was launched to offer comprehensive support tailored to the needs of LGBTQIA+ individuals within the Croatian public mental healthcare system.

**Objectives:**

The aim is to present a group processes, with a focus on the development of cohesion, within the BOJE programme - which includes group psychotherapy in structured three-month cycles and ongoing support groups.

**Methods:**

In October 2023, a multidisciplinary team at the University Psychiatric Hospital Sveti Ivan in Zagreb, Croatia, formed a Mental Health Protection Programme BOJE which consists of a counseling center, an outpatient clinic and a three-month therapeutic and educational cycle for LGBTQIA+ users. Over time, the programme has expanded to include support group, psychotherapeutic with families and a platform was provided for training healthcare professionals in LGBTQIA+ affirmative practice. In our experience, the programme’s main components are the three-month structured group cycle — combining psychoeducation, group psychotherapy and art therapy — and the ongoing support group.

**Results:**

To date, we have carried out more than 1,200 interventions — including counseling, psychiatric consultations, reviews, and psychotherapy. Four cycles of closed-group workshops have been completed with a very low dropout rate, and a fifth cycle is currently underway. The support group now brings together participants from all previous cycles. In both types of groups, we observed rapid development of cohesion, stronger interpersonal support, more active participation, better outcomes, and consistently low dropout rates.

**Conclusions:**

The BOJE programme was established in the Croatian healthcare system to address the impact of minority stress and support the mental health of LGBTQIA+ people. Our experience shows that group cohesion forms rapidly when participants share both an LGBTQIA+ identity and the programme framework. This reflects research demonstrating that shared identity can transform vulnerability into resilience, belonging, and mutual support.

**Disclosure of Interest:**

None Declared

